# Prevalence of tick-borne encephalitis virus in *Ixodes ricinus* ticks in northern Europe with particular reference to Southern Sweden

**DOI:** 10.1186/1756-3305-7-102

**Published:** 2014-03-11

**Authors:** John H-O Pettersson, Irina Golovljova, Sirkka Vene, Thomas GT Jaenson

**Affiliations:** 1 Medical Entomology Unit, Subdepartment of Systematic Biology, Department of Organismal Biology, Evolutionary Biology Centre, Uppsala University, Norbyvägen 18d, SE-752 36, Uppsala, Sweden; 2 Department of Virology, National Institute for Health Development, Tallinn, Estonia; 3 Public Health Agency of Sweden, Solna, Sweden

**Keywords:** *Ixodes ricinus*, Minimum infection rate, Real-time PCR, Sweden, Norway, Denmark, Finland, TBE, Tick-borne encephalitis virus, Virus prevalence

## Abstract

**Background:**

In northern Europe, the tick-borne encephalitis virus (TBEV) of the European subtype is usually transmitted to humans by the common tick *Ixodes ricinus*. The aims of the present study are (i) to obtain up-to-date information on the TBEV prevalence in host-seeking *I. ricinus* in southern and central Sweden; (ii) to compile and review all relevant published records on the prevalence of TBEV in ticks in northern Europe; and (iii) to analyse and try to explain how the TBE virus can be maintained in natural foci despite an apparently low TBEV infection prevalence in the vector population.

**Methods:**

To estimate the mean minimum infection rate (MIR) of TBEV in *I. ricinus* in northern Europe (i.e. Denmark, Norway, Sweden and Finland) we reviewed all published TBEV prevalence data for host-seeking *I. ricinus* collected during 1958–2011. Moreover, we collected 2,074 nymphs and 906 adults of *I. ricinus* from 29 localities in Sweden during 2008. These ticks were screened for TBEV by RT-PCR.

**Results:**

The MIR for TBEV in nymphal and adult *I. ricinus* was 0.28% for northern Europe and 0.23% for southern Sweden. The infection prevalence of TBEV was significantly lower in nymphs (0.10%) than in adult ticks (0.55%). At a well-known TBEV-endemic locality, Torö island south-east of Stockholm, the TBEV prevalence (MIR) was 0.51% in nymphs and 4.48% in adults of *I. ricinus*.

**Conclusions:**

If the ratio of nymphs to adult ticks in the TBEV-analysed sample differs from that in the *I. ricinus* population in the field, the MIR obtained will not necessarily reflect the TBEV prevalence in the field. The relatively low TBEV prevalence in the potential vector population recorded in most studies may partly be due to: (i) inclusion of uninfected ticks from the ‘uninfected areas’ surrounding the TBEV endemic foci; (ii) inclusion of an unrepresentative, too large proportion of immature ticks, compared to adult ticks, in the analysed tick pools; and (iii) shortcomings in the laboratory techniques used to detect the virus that may be present in a very low concentration or undetectable state in ticks which have not recently fed.

## Background

The common tick *Ixodes ricinus* is the most important arthropod vector of pathogens of human diseases in Europe [1,2]. One of these pathogens potentially causing human disease is the tick-borne encephalitis virus (TBEV), a member of the tick-borne group within the genus *Flavivirus*[3], family Flaviviridae [4]. Tick-borne encephalitis (TBE) is a potentially fatal disease syndrome of humans and some other mammals [5]. TBE is endemic in central, eastern, and northern Europe eastwards through Russian Siberia and China [6-8]. During the last two decades, 1990–2009, an annual mean incidence of 2,815 cases of human TBE was recorded for Europe, while a corresponding annual mean incidence of 5,682 human TBE cases was reported from Russia [7].

Currently, the TBEV complex is considered to encompass three virus subtypes; the European (TBEV-Eu), the Far-Eastern (TBEV-Fe), and the Siberian TBEV (TBEV-Sib) [4,5,9]. TBEV-Eu is mainly vectored by *I. ricinus* while *I. persulcatus* is the primary vector of the Siberian and Far Eastern subtypes [5]. The European subtype is present in certain foci in Sweden, Norway, Denmark, Finland, Russia, the Baltic countries and southwards through several east, central and south European countries [7]. The Far-Eastern subtype, in contrast to the Siberian subtype, has not yet been found in Northern Europe. However, it is present in populations of *I. persulcatus* in the Baltic area [10] and western Russia not far from the Finnish border. Its geographical range extends eastwards to China and Japan [9,11]. The Siberian subtype is found in Siberia, eastern Europe and western Russia [9,10,12], but also in Finland [13]. All three subtypes are known to co-circulate in areas where the geographical ranges of *I. ricinus* and *I. persulcatus* overlap [14,15]. The European subtype is the only subtype so far found in ticks in Sweden [16-18], Norway [19] and Denmark [20]. In Finland, both the European and Siberian viruses have been detected in *I. persulcatus*. Only the former virus subtype has been recorded from *I. ricinus* in Finland [13,21,22].

More than 70% of TBEV infections in humans are without symptoms [5]. Virulence and disease symptoms exhibit characteristic differences related to virus subtype. The overt disease caused by TBEV-Eu may range from a relatively mild influenza-like infection to a severe, life-threatening disease with paralytic long-lasting sequelae. The mortality rate caused by infections with TBEV-Eu is about 1–2% while that of the Siberian subtype rarely exceeds 8% [5]. The Far-Eastern subtype often causes a monophasic disease with a high rate of severe neurologic sequelae and a mortality rate that sometimes exceeds 20% [5,6,23-25].

In Sweden the first human TBE case was described in 1954 [26]. Four years later the virus was isolated from *I. ricinus* ticks and from a patient. Since then, the annual incidence of human TBE has increased from 60–80 cases/year before the 1990s to more than 100 cases/year since 2000, thereafter increasing even further to more than 150 cases/year since 2006 with a significant increasing trend during 2000–2012 [27]. This rise in TBE incidence in Sweden is attributed to a combination of biotic and climatological factors, particularly high abundance of roe deer and other cervids in southern Sweden since the mid-1980s and a warmer climate with a prolonged vegetation period [27,28]. Based on data for the year 2009 for the Scandinavian countries, Sweden has the highest TBE incidence (2.3 per 100 000), followed by Finland (0.5 per 100 000), Norway (0.2 per 100 000), and Denmark (0.02 per 100 000) [7]. The only regional estimates of TBEV prevalence in *I. ricinus* published so far refer to southwestern Sweden. They range from 0.10% to 0.42% [29].

Despite the great public health importance of TBE, some aspects of the ecology of TBEV have not been adequately investigated. One characteristic of the ecology of the TBE virus is its irregular distribution over a large geographical range with a patchy occurrence in restricted foci of limited size [30-33]. This is in contrast to several other *Ixodes*-transmitted pathogens, such as *Anaplasma phagocytophilum*[34,35] and some genospecies in the *Borrelia burgdorferi* sensu lato complex, the endemic regions of which are extensive and sometimes even include whole countries [36,37]. Another peculiarity of TBEV, which has puzzled scientists for a long time, is the low prevalence of the virus, usually <1%, in the *I. ricinus* population. This phenomenon also differs from the usually significantly higher prevalence of most of the bacteria vectored by *I. ricinus*[34,35,37,38]. Thus, the question arises how the virus can be maintained in a small focus for many years despite such apparently low infection prevalence in *I. ricinus*.

Here we present TBEV prevalence data based on virus screening of *I. ricinus* collected at 29 localities in the main TBEV-endemic regions of southern Sweden during 2008. We also provide a summary of all relevant, published TBEV-prevalence data for *I. ricinus* collected in Sweden and its three neighbouring countries Denmark, Norway and Finland.

## Methods

### Tick collection

**Figure 1:**
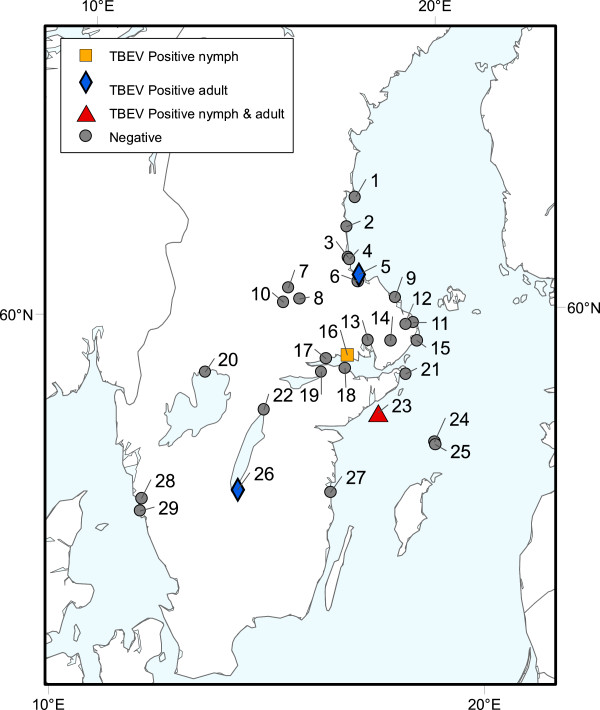
**Map of southern and central Sweden.** The numbers refer to localities where nymphs and adults of *Ixodes ricinus* ticks were collected. These ticks were subsequently analysed for TBEV infection. The name of each numbered locality and its GPS coordinates can be found in Table 1 and Additional file 1: Table S1, respectively.

Between May-September 2008, host-seeking (that usually do not contain any visible blood in the gut) *I. ricinus* were collected at 29 localities in southern and central Sweden (Figure 1, Additional file 1: Table S1) as previously described [39]. In short, a total of 2,074 nymphs and 906 adult ticks (481 females and 425 males) were collected by a person pulling a 1 × 1 (1 m^2^) white flannel cloth placed horizontally on the ground vegetation in deciduous or mixed deciduous/coniferous woodland biotopes [40]. At Norbo Finnmark, 12 adult *I. ricinus*, four of which were fully engorged, were removed from a pet dog (*Canis lupus domesticus*) (Table 1). All ticks were identified as *I. ricinus* based on morphological criteria according to [41,42]. The words “tick” and “ticks”, when used in this article, denote *I. ricinus*.

**Table 1:** **Summary of published and unpublished data on *I. ricinus *** **ticks collected in Sweden, Norway, Finland and Denmark analysed for TBE virus infection**

			**Number of collected ticks**					**Number of TBEV-positive**			**Prevalence estimate (%)**				
**Country**	**Collection year**	**Locality**	**Nymphs**	**Males**	**Females**	**Total**	**Pools**	**Positive pools**	**Positive nymphs**	**Positive adults**	**MIR nymphs**	**MIR adults**	**MIR all**	**Method**	**Reference**
Sweden	1958	96 km NE of Stockholm (9 sites)	35	898		933	24	4	1	3	2.86	0.33	0.42	MBI*	[43]
Sweden	2003	Torö	106	9		115	1	1	–	–	–	–	0.87	RT-PCR	[17]
Sweden	2003	Combined central Sweden (3 sites)	167	23		190	1	1	–	–	–	–	0.53	RT-PCR	[17]
Sweden	2006	3 sites south of Vänern (T1-T3)	4380	220	220	4820	263	11	9	2	0.21	0.45	0.23	RT-PCR	[29]
Sweden	2004	South-western Sweden (T4)	2740	70		2810	144	7	6	1	0.22	1.43	0.25	RT-PCR	[29]
Sweden	2008	Hudiksvall (1)	30	6	5	41	14	0	0	0	0	0	0	RT-PCR	This study
Sweden	2008	Stenö/Källskär (2)	300	90	92	482	202	0	0	0	0	0	0	RT-PCR	This study
Sweden	2008	Gävle (3)	4	1	0	5	2	0	0	0	0	0	0	RT-PCR	This study
Sweden	2008	Trödje (4)	3	2	2	7	4	0	0	0	0	0	0	RT-PCR	This study
Sweden	2008	Skutskär (5)	29	11	15	55	27	1	0	1	0	3.85	1.82	RT-PCR	This study
Sweden	2008	Älvkarleby (6)	10	6	13	29	20	0	0	0	0	0	0	RT-PCR	This study
Sweden	2008	Borlänge (7)	7	4	4	15	9	0	0	0	0	0	0	RT-PCR	This study
Sweden	2008	Vikmanshyttan (8)	15	2	4	21	7	0	0	0	0	0	0	RT-PCR	This study
Sweden	2008	Östhammar (9*)	94	10	15	119	31	0	0	0	0	0	0	RT-PCR	This study
Sweden	2008	Norbo Finnmark (10)	11	11	12	34	24	0	0	0	0	0	0	RT-PCR	This study
Sweden	2008	Väddö (11*)	32	10	6	48	18	0	0	0	0	0	0	RT-PCR	This study
Sweden	2008	Skebobruk (12*)	40	13	19	72	34	0	0	0	0	0	0	RT-PCR	This study
Sweden	2008	Morga (13*)	300	31	55	386	114	0	0	0	0	0	0	RT-PCR	This study
Sweden	2008	Rimbo (14*)	8	2	4	14	7	0	0	0	0	0	0	RT-PCR	This study
Sweden	2008	Kapellskär (15*)	373	59	70	502	151	0	0	0	0	0	0	RT-PCR	This study
Sweden	2008	Kolarvik (16*)	158	57	49	264	115	1	1	0	0.63	0	0.38	RT-PCR	This study
Sweden	2008	Västerås (17*)	137	11	24	172	47	0	0	0	0	0	0	RT-PCR	This study
Sweden	2008	Strängnäs (18*)	37	27	22	86	51	0	0	0	0	0	0	RT-PCR	This study
Sweden	2008	Eskilstuna (19*)	27	4	7	38	13	0	0	0	0	0	0	RT-PCR	This study
Sweden	2008	Karlstad (20)	9	5	3	17	9	0	0	0	0	0	0	RT-PCR	This study
Sweden	2008	Värmdö (21*)	46	3	6	55	16	0	0	0	0	0	0	RT-PCR	This study
Sweden	2008	Askersund (22)	36	0	5	41	9	0	0	0	0	0	0	RT-PCR	This study
Sweden	2008	Herrhamra (23*)	196	31	36	263	99	4	1	3	0.51	4.48	1.52	RT-PCR	This study
Sweden	2008	Kapellängen, GS (24) (P)	24	21	2	47	25	0	0	0	0	0	0	RT-PCR	This study
Sweden	2008	Gamla gården, GS (25) (P)	5	2	2	9	4	0	0	0	0	0	0	RT-PCR	This study
Sweden	2008	Jönköping (26)	30	2	1	33	5	1	0	1	0	33.33	3.03	RT-PCR	This study
Sweden	2008	Västervik (27)	14	4	5	23	10	0	0	0	0	0	0	RT-PCR	This study
Sweden	2008	Änggårdsbergen (28)	31	0	1	32	3	0	0	0	0	0	0	RT-PCR	This study
Sweden	2008	Särö Västerskog (29) (P)	68	0	2	70	8	0	0	0	0	0	0	RT-PCR	This study
Sweden	2008	Combined central Sweden, 12 sites*	1448	258	313	2019	258	5	2	3	0.14	0.53	0.25	RT-PCR	This study
Sweden	2008	Combined Sweden, 29 sites	2074	425	481	2980	1074	7	2	5	0.10	0.55	0.23	RT-PCR	This study
Sweden	1958–2008	Combined Sweden, 4 studies, 45 sites	9396	2337		11733	1510	30	18	11	0.19	0.47	0.26	*/PCR	[17,29,43], this study
Finland	1957–1960, 1964	Archipelago of southern-western Finland	4932	391	389	8131	249	18	–	–	–	–	0.22	MBI**	[44]
Finland	1957-1960, 1964	Southern Finland	124	9	0	133	7	0	–	–	–	–	0	MBI**	[44]
Finland	1957–1960, 1964	South-eastern Finland	1308	39	84	1643	51	1	–	–	–	–	0.06	MBI**	[44]
Finland	1996–1997	Isosaari (Mjölö) island, Helsinki	69	70		139	20	1	–	–	–	–	0.72	RT-PCR	[45]
Finland	1996–1997	Åland islands	203	247		450	48	1	–	–	–	–	0.22	RT-PCR	[45]
Finland	1996–1997	Helsinki city parks	74	123		726	130	0	–	–	–	–	0	RT-PCR	[45]
Finland	2004	Kokkola (Karleby) archipelago (10 sites)	72	539	570	1181	122	13	–	–	–	–	1.10	RT-PCR	[13]
Finland	2003	Kumlinge	–	–	–	454	46	4	–	–	–	–	0.88	RT-PCR	[21]
Finland	2005	Isosaari (Mjölö) island, Helsinki	–	–	–	96	11	1	–	–	–	–	1.04	RT-PCR	[21]
Finland	2007	Turku (Åbo) archipelago	–	–	–	1039	315	1	–	–	–	–	0.10	RT-PCR	[21]
Finland	2005	Lappeenranta (Villmanstrand)	–	–	–	292	29	2	–	–	–	–	0.68	RT-PCR	[21]
Finland	2008	Närpiö (Närpes)	–	–	–	36	–	0	–	–	–	–	0	RT-PCR	[21]
Finland	1957–2008	Combined Finland, 4 studies, ≥ 27 sites				14320	2490	42	–	–	–	–	0.29	**/PCR	[13,21,44,45]
Norway	2003	Vest-Agder and Hordaland county	360					1	–	–	–	–	0.28	RT-PCR	[19]
Norway	2004	Vest-Agder and Hordaland county	450					1	–	–	–	–	0.22	RT-PCR	[19]
Norway	2009	Risør, Dalen (S1)	900	–	–	900	90	1	1	–	0.11	–	0.11	RT-PCR	[46]
Norway	2009	Arendal (S2–S3)	1350	–	–	1350	135	8	8	–	0.59	–	0.59	RT-PCR	[46]
Norway	2009	Mandal (S4–S5)	1520	–	–	1520	152	9	9	–	0.59	–	0.59	RT-PCR	[46]
Norway	2009	Lyngdal (S6–S7)	1860	–	–	1860	186	6	6	–	0.32	–	0.32	RT-PCR	[46]
Norway	2003–2009	Combined Norway, 2 studies, 9 sites				6440	≥ 563	26	–	–	–	–	0.40	RT-PCR	[19,46]
Denmark	1999	Bornholm (7 sites)	3843	215		4058		2	–	–	–	–	0.05	RT-PCR	[47]
Denmark	2002–2003	Northern Zealand	50	25	30	105	3	1	1	–	2.00	–	0.95	RT-PCR	[48]
Denmark	2002–2003	3 different sites	62			62	9	0	–	–	–		–	RT-PCR	[48]
Denmark	2011	Tokkekøb (3 sites, Jun.)	854	22	20	896	24	3	2	1	0.23	2.38	0.33	RT-PCR	[20]
Denmark	2011	Tokkekøb (3 sites, Sept.)	700	15	15	730	8	5	5	0	0.71	–	0.68	RT-PCR	[20]
Denmark	2011	Grib Forest	183	9	6	198	13	0	0	0	–	–	–	RT-PCR	[20]
Denmark	2011	Bornholm (3 sites)	738	37	41	816	13	0	0	0	–	–	–	RT-PCR	[20]
Denmark	1999–2011	Combined Denmark, 3 studies , ≥ 18 sites				6865	≥ 70	11	–	–	–	–	0.16	RT-PCR	[20,47,48]
**All four countries**	**1957–2011**	**All sites (≥ 99) included in references**				**39358**	**3171**	**109**					**0.28**		[13,17,19-21,29,43-48], this study

Numbers within parenthesis for the present study correspond to sampling localities in Figure 1.

MIR, Minimum Infection Rate (%). P, adult ticks were pooled.

MBI*, Mouse brain inoculation and tissue cultures followed by neutralization tests and complement fixation tests [43].

MBI**, Mouse brain inoculation followed by haemagglutination and haemagglutination-inhibition tests [44].

### RNA extraction and detection of TBEV

RNA was extracted, amplified and screened for TBEV in nymphs and adults of *I. ricinus* using a Real-Time Reverse Transcription Polymerase Chain Reaction (RT-PCR) targeting a certain region in the 3′-terminal of the TBEV genome modified after Schwaiger and Cassinotti [49] as previously described for the detection of TBEV in nymphs [50] and adult [29]*Ixodes* ticks, respectively. Each RNA extraction was made from a pool of ~20 nymphs, or a single adult tick, except for adult ticks collected at Gotska Sandön and Särö Västerskog, which were pooled as shown with the letter P in Table 1.

### Statistical analyses

The prevalence of TBEV infection in *I. ricinus* ticks of a certain stage collected at a certain locality was estimated using the Minimum Infection Rate (MIR), i.e. the minimum infected proportion expressed as a percentage:

$$\text{MIR}=\left(p/N\right)\times 100\%$$

where:

p = the number of positive pools

N = the total number of ticks tested

The MIR is considered acceptable for the present type of data on arboviruses occurring in their vector populations at low prevalences [51-53]. This method assumes that only one infected tick is present in each positive pool [51]. The MIR also permits comparison of prevalence estimates from different investigations in which different tick collection strategies were used, and where the number of positive pools and the total number of ticks analysed are known. Fisher’s exact test was used to test if there is a significant difference, based on a two-tailed hypothesis, between two MIR estimates.

### Gathering of TBEV prevalence data from previous studies

TBEV prevalence data were included in our review if the study reported at least the total number of ticks and/or tick stage(s) collected, and the total number of TBEV positive pools and/or individual ticks. We included only publications presenting TBEV-analyses of ticks collected in Denmark, Finland, Norway or Sweden.

## Results

### TBEV in nymphs or adult ticks in Sweden

A total of 2,074 nymphs and 906 adults of *I. ricinus* were collected from 29 study localities in Sweden during 2008 (Figure 1). Among 108 pools of nymphs tested two pools were TBEV-positive, as indicated by RT-PCR (Table 1): One pool originated from Kolarvik and the other from Herrhamra. Five of 906 adult ticks tested individually were TBEV-positive by RT-PCR (Figure 1, Table 1): One tick originated from Jönköping, three ticks from Herrhamra on the island of Torö, and one from Skutskär. The MIR calculated was 0.10% for the nymphs and 0.55% for the adult females (Fisher’s test: P = 0.030). Four of 7 TBEV-positive ticks originated from the same small island, Torö, which is a well-known TBEV-endemic focus. At Torö, we detected the TBEV infection in both nymphs (MIR = 0.51%) and adults of both sexes (MIR = 4.48%) of *I. ricinus* (Fisher’s test: P = 0.0521).

Based on all nymphs and adults of *I. ricinus* from the 29 localities the TBEV prevalence, calculated as a MIR, was 0.23% (7 positive pools; 1,007 negative pools; N = 2,980 ticks analysed). For ticks collected in the northern part of southern Sweden (Eskilstuna, Herrhamra, Kapellskär, Kolarvik, Morga, Rimbo, Skebobruk, Strängnäs, Väddö, Värmdö, Västerås, Östhammar) (Figure 1, Table 1), the MIR was 0.25%. This infection prevalence comes from 5 positive pools (2 nymphal pools and 3 adult ticks; 84 negative nymphal pools and 568 negative specimens) out of 2,019 ticks tested (1,448 nymphs and 571 adults).

### TBEV in ticks from the four countries

The overall mean MIR estimate for TBEV in *I. ricinus* for the four neighbouring countries, Denmark, Sweden Norway and Finland, was 0.28% (109 TBEV-positive pools of 39,358 ticks tested, Table 1), which corresponds to approximately one TBEV-positive tick in each sample of 360 ticks. However, it should be noted that this is an overall mean MIR for the four countries and is based on both nymphs and adult ticks. The reason for combining these life stages is that in several of the publications analysed information about the tick stage(s) analysed was not stated. In the total data set (Table 1), the nymphal to adult ratio is approximately 5:1. This is within the range of the ratio of nymphs to adults that can be found in research on population ecology of *I. ricinus*[40,54-56].

## Discussion

### TBEV prevalence in Sweden and neighbouring Nordic countries

The overall mean TBEV prevalence for *I. ricinus* in the four Scandinavian countries was 0.28%. This corresponds to almost one TBEV-positive specimen in each sample of 360 ticks collected. It should be emphasised that the latter percentage, 0.28%, for Scandinavia refers to a mixture of pools containing both nymphs and adult ticks. It is well known that the infection prevalence of adult female ticks is usually significantly higher than that of nymphs [57]. This is most likely mainly due to the fact that, during their development from larva to adult tick, the questing adult tick female has usually blood-fed twice, i.e. on two different, potentially TBEV-infected host individuals. In contrast, the questing nymphs have fed only once [58,59]. This is also indicated in the present study by the data from Herrhamra where the MIR was 0.51% for nymphs and 4.48% for adults. Thus, if we had analysed relatively more adult ticks from Herrhamra it is likely that the overall TBEV infection prevalence estimate would have appeared even higher. The estimated mean TBEV prevalence is similar to those estimated for another *I. ricinus-*transmitted pathogen, *B. miyamotoi*, in Sweden [60] and Estonia [50] but lower than those usually recorded for other pathogens vectored by *I. ricinus*, such as *B. afzelii, B. garinii* and *B. valaisiana*[37,60], and *A. phagocytophilum*[34,35,39,61].

The estimated infection prevalence increased when the TBEV analysis was restricted to ticks collected only from one locality, Herrhamra on the island of Torö. This is a well-known TBEV-enzootic area, where many people have contracted neuroinvasive TBE. The island seems to be an example of such a focus, as described by Dobler and co-workers [33] in which the TBEV occurs permanently within a restricted geographical area. Consequently, if a larger number of ticks had been collected outside of the TBEV focus and had been included in the virological analysis the TBEV prevalence estimate would have been reduced. Furthermore, another obvious problem with the use of the MIR estimate on pooled samples occurs when ticks are collected in a habitat where the infection rate is relatively high. Here, several virus-infected tick specimens could be present in one pool; yet, such a positive pool would be considered to contain only one infected tick, thereby reducing the prevalence estimate to fall below the actual prevalence [51-53].

### Maintenance of TBEV in nature

The TBE virus is maintained and transmitted in natural foci mainly in five ways: (i) by ticks becoming infected when feeding on viraemic hosts whereby infective ticks, in a subsequent stage, may transmit the virus to susceptible, new hosts; (ii) by transovarial transmission in ticks; (iii) by transstadial transmission in ticks; (iv) by sexual transmission from a male tick to a female tick; and (v) by non-viraemic transmission from infective tick(s) co-feeding adjacent to susceptible ticks on a non-infected and/or non-viraemic host [62-65].

Transmission of the TBEV can take place when tick larvae or nymphs feed on (I) viraemic *Apodemus* mice or *Myodes* voles. *Apodemus* mice are regarded as the optimal transmission hosts for this mode of TBEV transfer, since they do not rapidly become resistant to the feeding ticks [66]. This is in contrast to bank voles, which rapidly become resistant to the feeding ticks [67]. Furthermore, it is generally accepted that any viraemia in rodents, infective to feeding ticks, will only last for a few days. Therefore, this mode of TBEV transmission is not considered sufficiently effective to solely maintain the virus in the *I. ricinus* populations [65,68,69]. Still, rodents can act as TBEV reservoirs since TBEV can be detected in infected rodents for periods of several months, including during the winter period [70,71].

Even ticks act as reservoirs for the TBEV due to their capacity of transovarial and transstadial transmission. Once infected, the tick will usually remain infected throughout its life [65]. However, transovarial transmission only occurs at a low frequency and is, therefore, on its own considered not sufficiently effective to maintain TBEV in the vector population [72]. Sexual transmission occurs when TBEV-infected tick males infect females by transferring infectious saliva and/or seminal fluid during copulation [73]. It is not known if transovarial and sexual transmission are necessary for the long-term persistence of the virus in the ecosystem. Possibly, they may have evolved to function as auxiliary modes of transmission by which the TBEV can ‘survive’ in the ecosystem during periods when the availability of vertebrate virus transmission hosts and vertebrate virus reservoirs are unavailable for the questing ticks to feed on. Non-viraemic transmission is generally regarded as the main mode of transmission by which TBEV is transmitted to infectible ticks and maintained in nature. Non-viraemic transmission may occur when one or more susceptible ticks are feeding in close proximity to an infective tick [62,65,68,74]. In this way, transmission of TBEV takes place when infective ticks, typically nymphs, are feeding on the host. TBE virions will be transferred with the saliva, which is injected by the blood-feeding, virus-infective nymphs into the feeding site. Here, virions may be phagocytosed by leukocytes. Some of these virus-infected blood cells may then be ingested by susceptible ticks, typically larvae, which in this manner become infected [62]. It should be noted that for virus transmission to occur among co-feeding ticks it is not necessary that a viraemia is present in the host [63]. However, synchronous questing activity of infective ticks and susceptible ticks is necessary for the TBE virus to be transmitted in this way [75]. Non-viraemic transmission supported by a low degree of transovarial transmission is considered sufficient to maintain the TBEV at the prevalence levels at which it generally occurs in *I. ricinus*[76].

There is some evidence that goats are not competent hosts either for viraemic or non-viraemic transmission of TBEV among co-feeding ticks [77]. However, to our knowledge, there exists no experimental evidence that cervids are incompetent hosts for non-viraemic transmission of TBEV among co-feeding ticks. Although the TBEV viraemia in deer may be of a short duration and of insufficient magnitude in cervids we should not yet reject the possibility that co-feeding transmission via non-viraemic cervids might take place. In TBE-endemic areas both domesticated and wild ungulates, especially roe deer, usually have antibodies to TBEV [78] and the seroprevalence in TBEV foci can be high in such mammals [77]. Labuda and co-workers demonstrated that natural hosts, which have neutralizing antibodies to the TBEV and apparently are immune to TBEV (i.e., without any viraemia) still can support transmission of this virus from infective to uninfected ticks feeding close together on the same host [63]. All stages of *I. ricinus* preferentially attach to the neck and head region of roe deer and both larvae and nymphs occur at the highest densities on the head of this important tick maintenance host [79]. These facts support the idea that the roe deer is one of the most important host species for adult *I. ricinus* ticks. These facts also support the notion that roe deer possibly can support the non-viraemic transmission of TBEV to uninfected ticks. Indeed, roe deer abundance may be a useful indicator of the risk for people in TBEV-endemic areas to contract a TBE virus infection. Along these lines, Zeman and Januska [80] showed that the risk of TBE was associated with the abundance of roe deer and mice (*Apodemus* spp.).

### Is the TBEV prevalence in the tick population unexpectedly low?

Two important questions are: (I) Is the infection prevalence of TBEV in the *I. ricinus* populations exceptionally low? (II) How can the virus persist in nature despite such ‘low’ infection prevalence? Prevalence rates of TBEV in *I. ricinus* populations in endemic areas usually range from 0.1–5% [7,10,57,81] and the prevalence usually fluctuates from year to year and among regions [57]. It is likely that both viraemic and non-viraemic transmission of TBEV to uninfected ticks occur more frequently during years of peak abundance of small mammals [27]. So these fluctuations in TBEV infection prevalence are presumably to some degree due to the varying densities of reservoir-competent *vs.* reservoir-incompetent tick hosts. Both TBEV and *B. miyamotoi* seem to have geographical distributional ranges composed of a patchwork of relatively small enzootic foci. Here, both pathogens seem to be present at low prevalences in their invertebrate reservoir and vector, i.e. *I. ricinus*. Both pathogens rely, to a small extent, on transovarial transmission. It might be a trait, which has evolved in TBEV and in *B. miyamotoi,* to enable these human pathogens to ‘survive’ independent from vertebrate transmission hosts during periods when the availability of such tick hosts, i.e. small mammals, is low or non-existent.

One reason for the low *apparent* prevalence recorded in many investigations may be due to inclusion of ticks from non-endemic areas adjacent to the relatively confined TBEV-infected foci [33]. If the limits of such a focus are known and ticks are collected only from within the borders of this TBEV focus, the virological analysis of these ticks is likely to give a higher TBEV prevalence estimate than if ticks from outside the TBE focus were included in the analysis.

It has been known for many years that TBEV infection rates of blood-fed ticks, collected from humans or other hosts, are usually higher than those of unfed, questing ticks collected from the vegetation in the same area [81,82]. In a series of experiments, it was shown that TBEV-infected ticks become more active in their host-searching behaviour compared to that of uninfected ticks [83,84]. It was also suggested that TBEV might occur in undetectable concentrations in infected ticks in nature, and that it is not until the tick is feeding, that virus quantities can increase 100-fold [83] so that TBEV becomes detectable [84]. It may be that the virus occurs in an undetectable, seemingly ‘latent’ state, in the host-seeking TBEV-infected tick. Components in the blood and/or the increased temperature might be triggering immature virions to become mature virions. Another possibility is that the amount of virions in the non-blood-fed tick is below the detection limit of the methodology ordinarily used. Different methods for detecting viruses and microorganisms can have different sensitivities [85,86]. Thus, it has been emphasized that if the sensitivity of the PCR-based detection method used is not optimal, it is likely that the infection prevalence will be underestimated [57]. The PCR method that we used, which is a modification of the method described by Schwaiger and Cassinotti [49], has a detection limit of 1–10 copies per reaction. Therefore, the TBEV prevalences of the ticks collected in Sweden and analysed by us, are most likely not underestimated.

The observed, relatively low TBEV prevalence in *I. ricinus* in nature is likely explained by a combination of such factors as just mentioned. Future studies should aim to explain in more detail the relative importance of the different environmental, pathogen-, tick-, and vertebrate-related factors, which are necessary for an area to be a long-term TBEV enzootic focus.

## Conclusions

If the ratio of nymphs to adult ticks in the TBEV-analysed sample differs from that in the *I. ricinus* population in the field, the MIR obtained will not necessarily reflect the TBEV prevalence in the field. The relatively low TBEV prevalence in the potential vector population recorded in most studies may partly be due to: (i) inclusion of uninfected ticks from the ‘uninfected areas’ surrounding the TBEV endemic foci; (ii) inclusion of an unrepresentative, too large proportion of immature ticks, compared to adult ticks, in the analysed tick pools; and (iii) shortcomings in the laboratory techniques used to detect the virus that may be present in a very low concentration or undetectable state in ticks which have not recently fed.

## Competing interests

The authors declare that they have no competing interests.

## Authors’ contributions

JP and TJ collected, reviewed, analysed and synthesised published and unpublished information for this article; JP and TJ wrote the initial and final versions of the manuscript. JP and TJ collected ticks in the field that were analysed for TBEV infection by JP, IG and SV in the laboratory of the Public Health Agency of Sweden (formerly the Swedish Institute for Communicable Disease Control), Solna, Sweden. All co-authors co-revised the manuscript and co-refined the intellectual content of the manuscript. All authors read and approved the final version of the manuscript.

## Supplementary Material

Additional file 1: Table S1 Name and GPS coordinates for each locality where ticks were collected. Numbers refer to the same numbers in Figure 1 and Table 1. P = adult ticks were pooled. Click here for file
