# Supplementary material for: Prevalence of tick-borne encephalitis virus in Ixodes ricinus ticks in northern Europe with particular reference to Southern Sweden
Source: Parasit Vectors. 2014 Mar 11;7:102. doi: 10.1186/1756-3305-7-102 (PMC4007564; doi:10.1186/1756-3305-7-102)
Supplement: Additional file 1: Table S1 — Name and GPS coordinates for each locality where ticks were collected. Numbers refer to the same numbers in Figure 1 and Table 1. P = adult ticks were pooled. [file 1756-3305-7-102-S1.docx]

**Table S1 Name and GPS coordinates for each locality where ticks were collected. Numbers refer to the same numbers in Figure 1.**

| **GPS coordinates** | | | | |
| --- | --- | --- | --- | --- |
| **#** |  | **Locality** | **LATITUDE** | **LONGITUDE** |
| 1 |  | Hudiksvall | 61,6372963 | 17,4464491 |
| 2 |  | Stenö/Källskär | 61,2535660 | 17,1885400 |
| 3 |  | Gävle | 60,8506019 | 17,1951389 |
| 4 |  | Trödje | 60,8259167 | 17,2384444 |
| 5 |  | Skutskär | 60,6225000 | 17,4671019 |
| 6 |  | Älvkarleby | 60,5335000 | 17,4389676 |
| 7 |  | Borlänge | 60,4677778 | 15,5894444 |
| 8 |  | Vikmanshyttan | 60,3223565 | 15,8886204 |
| 9 |  | Östhammar | 60,2984769 | 18,4109306 |
| 10 |  | Norbo Finnmark | 60,2811111 | 15,4547222 |
| 11 |  | Väddö | 59,9595509 | 18,8499583 |
| 12 |  | Skebobruk | 59,9447546 | 18,6482130 |
| 13 |  | Morga | 59,7577778 | 17,6480093 |
| 14 |  | Rimbo | 59,7402870 | 18,2275370 |
| 15 |  | Kapellskär | 59,7202824 | 18,9147917 |
| 16 |  | Kolarvik | 59,5755556 | 17,0950000 |
| 17 |  | Västerås | 59,5380556 | 16,5386111 |
| 18 |  | Strängnäs | 59,4026806 | 17,0283750 |
| 19 |  | Eskilstuna | 59,3611296 | 16,4171620 |
| 20 |  | Karlstad | 59,3589259 | 13,4355694 |
| 21 |  | Värmdö | 59,2958333 | 18,5817546 |
| 22 |  | Askersund | 58,8813519 | 14,9522315 |
| 23 |  | Herrhamra | 58,8070231 | 17,8255556 |
| 24 |  | Kapellängen, GS (P) | 58,3872685 | 19,2005093 |
| 25 |  | Gamla gården, GS (P) | 58,3500741 | 19,2168472 |
| 26 |  | Jönköping | 57,8301481 | 14,3044444 |
| 27 |  | Västervik | 57,7876528 | 16,5827593 |
| 28 |  | Änggårdsbergen | 57,6774074 | 11,9501296 |
| 29 |  | Särö Västerskog (P) | 57,5138889 | 11,9282407 |
